# Clinical outcomes of robotic-assisted laparoscopic partial nephrectomy with renal hypothermia perfusion by renal artery balloon catheter in treating patients with complex renal tumors

**DOI:** 10.3389/fonc.2022.918143

**Published:** 2022-08-26

**Authors:** YuChen Bai, YunKai Yang, HaiBin Wei, Jing Quan, Fei Wei, Qi Zhang, Feng Liu

**Affiliations:** ^1^ Urology and Nephrology Center, Department of Urology, Zhejiang Provincial People’s Hospital, Hangzhou Medical College, Hangzhou, China; ^2^ Graduate Department, Bengbu Medical College, Bengbu, China

**Keywords:** RALPN, renal artery balloon catheter, complex renal tumor, hypothermic perfusion, GFR

## Abstract

**Objective:**

This study aimed to investigate the safety and efficacy of renal hypothermic perfusion by renal artery balloon catheter during robot-assisted laparoscopic partial nephrectomy (P-RALPN) for patients with complex renal tumors.

**Materials and methods:**

We retrospectively identified 45 patients with complex renal tumors who received standard robot-assisted laparoscopic partial nephrectomy (S-RALPN) and 11 patients treated with P-RALPN from September 2017 to October 2021. Preoperative patients’ characteristics and intraoperative surgical parameters including operating time, blood loss, hospitalization, pre- and post-surgical glomerular filtration rate (GFR), and postoperative survival time were collected and compared between the two groups. The patients’ body temperature, real-time kidney temperature, and short-term renal function were analyzed in the P-RALPN group.

**Results:**

There was no statistically significant difference on median intraoperative estimated blood loss and postoperative hospitalization between the two groups. Patients who received P-RALPN had a slightly longer operative time than those who received S-RALPN (103.1 versus 125.9; *p* = 0.09). In the P-RALPN group, the volume of perfusion solution was 533.2 ml (range, 255.0–750.0 ml), the median temperature of kidney was 22.6°C (range, 21.7–24.1°C) after the kidney cools down, and the median minimum intraoperative temperature of patients was 36.1°C (range 35.2–36.7°C). The ischemia time in the S-RALPN group was markedly lower than that in the P-RALPN group (21.5 versus 34.8; *p* < 0.01). However, the loss of GFR was much higher for the S-RALPN group after the surgery. (28.9 versus 18.4; *p* < 0.01). Importantly, patients had similar postoperative survival time between the two groups (*p* = 0.42; HR = 0.27).

**Conclusion:**

P-RALPN is a safe and feasible surgery in the treatment of patients with complex renal tumors, which provides a new operative approach for clinicians to treat these patients.

## Introduction

Renal tumor accounts for appropriately 2%–3% of adult tumors. More than 80% of them are malignant tumors (1, 2). Nephron-sparing partial nephrectomy has become one of the standard-of-care approaches for treating patients with renal tumors. In the past, clinicians often performed open partial nephrectomy (OPN). With the development of laparoscopic technology, laparoscopic partial nephrectomy (LPN) has replaced OPN as the standard treatment for localized renal tumors (3–8). Although LPN has significantly improved the overall prognosis of patients with renal tumors, it could simultaneously increase the risk of postoperative chronic kidney disease, such as long-term postoperative hematuria, infection, and renal insufficiency (9–11).

Recently, robot-assisted laparoscopic technology has been demonstrated as a qualitative operation. Robot-assisted laparoscopic partial nephrectomy (RALPN) has many advantages, including a three-dimensional (3D) surgical field enabling much more precise tumor resection and a better foundation for challenging and complex operations. Previously, several studies reported that RALPN had less complications than traditional surgical approaches, which has been gradually accepted by clinicians (12–14). However, when we treated patients with complex renal tumors (for example, multiple renal tumors, giant hamartomas, and endophytic tumors of the renal hilum), surgical resection requires a longer time and sometimes even reconstruction of the kidney structure. The kidney has a rich blood supply, and these operations need to be performed under renal artery blocked conditions. Hence, renal function may be damaged due to the prolonged ischemia time during operation (15–17). In this case, it is also difficult to remove very complex renal tumors in a short time.

How to preserve the renal function of patients after partial nephrectomy is a big issue in clinical practice. In the past, methods used included perirenal ice-water cooling technology, retrograde ureteral hypothermic perfusion technology, and local ice cryogenic method. These methods cannot effectively reduce the temperature of renal parenchyma, or the local temperature was too low, thus resulting in kidney injury due to freezing (18–21). The cold perfusion technology breaks the limitation of warm ischemia time (WIT) when RALPN was conducted to remove complex renal tumors. It could reduce the temperature of renal parenchyma in a short time, thus protecting the residual kidney function. However, they cannot stably maintain the low-temperature environment of the kidney during the operation, resulting in a transient warm ischemia state of the kidney during the operation (22). Since 2018, our department began to conduct a novel surgical procedure: inserting a balloon catheter through the renal artery, perfusing with low-temperature sodium lactate Ringer’s solution with a similar blood osmotic pressure and density, and using a stable power pump system to continuously maintain the kidneys in a “cold ischemia” state of hypothermia and ischemia; with the assistance of the Da Vinci robot, complex renal tumors can be quickly and safely resected. In this study, we firstly reported the safety and efficacy data of renal hypothermic perfusion by renal artery balloon catheter during robot-assisted laparoscopic partial nephrectomy (P-RALPN) for treating patients with complex renal tumors.

## Materials and methods

### Study population

We identified 45 patients from 778 patients with renal tumors who received standard robot-assisted laparoscopic partial nephrectomy (S-RALPN), and 11 patients received P-RALPN from September 2017 to October 2021 in our hospital. In order to accurately measure the unilateral renal function and total renal function, all eligible patients would receive the renal ECT (emission computed tomography) as routine examination preoperatively and 3 months post-operation. As a “gold standard” indicator for measuring renal function, glomerular filtration rate (GFR) was also recorded. GFR was defined as the amount of filtrate produced by the two kidneys per unit time, which could accurately describe renal function. The inclusion criteria were as follows: (1) warm ischemia time >30 min; (2) R.E.N.A.L. score of more than 8 (the R.E.N.A.L. nephrometry score: A Comprehensive Standardized System for Quantitating Renal Tumor Size, Location and Depth), consisting of radius (tumor size as maximal diameter), exophytic/endophytic properties of the tumor, nearness of the tumor’s deepest portion to the collecting system or sinus, anterior (a)/posterior (p) descriptor, and the location relative to the polar line; and (3) patients who have no mental illness and able to tolerate treatments. The exclusion criteria were as follows: (1) warm ischemia time <30 min; (2) renal score less than 8; and (3) patients who have severe lung or heart disease and who were not able to perform the interventional operation. Preoperative patients’ age, sex, smoking history, body mass index (BMI), ASA score, laterality, chronic disease, mass location, R.E.N.A.L. score, intraoperative surgical parameters including operating time, blood loss, hospitalization, pre- and post-surgical glomerular filtration rate (GFR), and postoperative survival time were collected and compared between the two groups. Age and smoking status were recorded at initial diagnosis. A never smoker was defined as a person who had smoked <100 cigarettes during his/her lifetime. The patients’ body temperature, real-time kidney temperature, and short-term renal functional were analyzed in the P-RALPN group. In order to accurately measure the unilateral renal function and total renal function, all eligible patients would receive renal emission computed tomography (ECT) as routine examination preoperatively and 3 months post-operation. One experienced surgeon in RALPN performed all surgeries. The study protocol was approved by the ethics committee and institutional review board of our hospital. Patients who met the above-mentioned criteria were included. We collected the data of eligible patients from electronic medical records by using the same requirements for clinical data.

### Surgical technique

**Figure 1:**
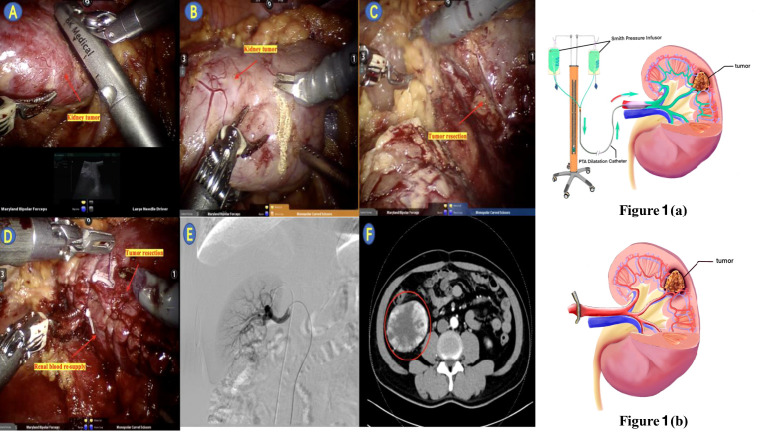
The schematic diagram of the P-RALPN shown as **(a)** and S-RALPN shown as **(b)**; **(A)** Ultrasound located the tumor intra-operation; **(B)** Balloon perfusion began, renal artery occlusion; **(C)** Tumor resection; (D) Renal artery occlusion completed and renal blood re-supply; **(E)** Pre-operative angiography confirmed the presence of renal arteriovenous ducts; **(F)** Pre-operative enhanced CT results of patient.

(1) Renal artery balloon catheter placement: the patient underwent preoperative renal artery catheterization pre-operation, the patient was routinely disinfected at the groin area, and the procedure was performed in the intervention room. The right femoral artery was punctured with the modified Seldinger technique, and a 5F catheter with its sheath was inserted into the right renal artery DSA, making the renal tumor visible, and the 5 mm × 4 cm balloon was replaced through the guide wire. The representative result after renal artery balloon catheter placement is shown in **Figure 1A.**

(2) Power maintenance perfusion: the range of perfusion pressure in the human kidney was 80–180 mmHg; the blood flow velocity in the renal artery was about 80–120 cm/s; the inner diameter of the renal artery is about 0.25 cm, and the intra-arterial pressure F = 0.2 N. We have two infusion boosters with a maximum height of 300 mmHg, combined with an infusion tower with a maximum height of 2.75 m, to form a power system for the perfusate.

(3) P-RALPN: taking the left kidney as the example, we have transferred the patient from the intervention room to the operation room directly after renal artery balloon catheter placement. After successful anesthesia, the patient was placed in the right lateral decubitus position. The skin was incised 2 cm above the iliac spine. The perirenal fat was pushed away after touching the lower pole of the kidney with fingers, and the peritoneum was pushed to the ventral side. The volume of the artificial balloon was 900 ml after expansion of abdominal cavity. A 1.0-cm Trocar was introduced and fixed with sutures. After successfully diagnosing pneumoperitoneum (pressure, 14 mmHg), 8-mm and 8-mm Trocars were placed under the costal margin of the midaxillary line and the posterior axillary line, respectively, under laparoscopic monitoring. The observation holes are opened with three transverse fingers, and a 12-mm Trocar was placed. The robotic laparoscopic surgical instruments are respectively introduced. The prerenal space and postrenal space fat, Gerota space, free peritoneum, and free perirenal fat were separated with electrocautery, and the middle and lower poles of the left kidney were continued to be exposed. The left gonadal vein was closed with Bulldog clamps. Saline (1.5 ml) was injected to inflate the balloon in order to fix the catheter and occlude the renal artery. An ultrasound probe was used to check blood flow and locate the edge of the tumor during operation and ensure that blood flow has stopped completely (**Figure 1B**). The color of the kidney surface turned to white after blocking the renal artery (**Figure 1C**). A positive pressure infusion bag was used to perfuse the lactated Ringer’s solution at 4°C. Bulldog clips are used to close the distal end of the renal vein (near the inferior vena cava side) in order to prevent fluid from entering systemic circulation and to open the gonadal veins as outlets for perfusion solutions. We chose the gonadal vein route for draining the fluid because of its advantages: (1) if we cut open the renal vein for drainage, we will have to perform vascular anastomosis after the renal tumor is removed, which will take longer and may result in a larger damage than *via* the gonadal vein; (2) the anatomy of the gonadal vein is easier to find during the operation; and (3) we just need a vascular clip to close the gonadal vein, which could save time, since vascular anastomosis is no longer necessary. Turn on the power system, start rapid perfusion to the affected kidney, rapidly perfuse nearly 200 ml of 4°C sodium lactate Ringer’s liquid into the kidney within 2 min through the catheter, and clamp the catheter immediately after the rapid perfusion. Monitor the surface temperature of the renal parenchyma in real time with a thermometer probe, as well as the patient’s body temperature. The external pressure and the flow rate of the intravascular perfusion fluid are adjusted when the temperature of the kidney drops. Afterwards, slow perfusion was started in order to maintain the kidney in a hypothermic and ischemic environment. Complete tumor resection: the wound was sutured with 3-0 and 2-0 absorbable sutures at multiple layers (**Figure 1D**). The balloon on the catheter was drained by injector after the renal tumor wound suture was completed. Then, the renal artery was opened to observe the blood supply of the renal parenchyma (**Figure 1E**). A perirenal drainage tube was settled. The renal artery balloon was removed, the femoral artery was compressed with sandbags, and the patient was returned to the postoperative intensive care unit. The schematic diagram of the P-RALPN and S-RALPN is shown in **Figure 1**. The per-operation CT of one patient is shown in **Figure 1F**.

### Statistical analysis

SPSS version 24.0 and GraphPad Prism version 9.1 were used to perform statistical analyses. Our research and data analysis follow the STROBE guidelines. Classification data are collected and analyzed as numbers and percentages. Student’s *t*-test was used for quantitative data, the *χ* ^2^ test or Fisher’s exact test was used for count data, and multiple regression analysis was used for multiple continuous and dependent variables. Two-sided *p* < 0.05 was considered as a statistically significant difference.

## Results

### Patients’ cohort

From September 2017 to October 2021, we retrospectively identified 56 patients with complex renal tumors, including 45 (80%) patients who received S-RALPN and 11 (20%) patients who received P-RALPN. The median age of S-RALPN and P-RALPN was 59.5 years (range, 14.0–76.0 years) and 64.6 years (range, 45.0–87.0 years), respectively. Most of the patients were male (78.6%), and the American Society of Anesthesia (ASA) score was low (one to two points; 92.8%). Patients’ characteristics are summarized in **Table 1**. The distribution of age, sex, smoking history, body mass index (BMI), ASA score, laterality, chronic disease, mass location, and R.E.N.A.L. score was comparable between two groups. The tumors in both groups were mostly located in the middle of the kidney, and the R.E.N.A.L. score was high (10–12 points; 64%).

**Table 1:** Patient characteristics.

	S-RALPN, *n* (%)	P-RALPN, *n* (%)	*p*
No. of patients	45.0	11.0	
Median age, years (IQR)	59.5 (14.0–76.0)	64.6 (45.0–87.0)	0.24
Male	37.0 (82.0)	7.0 (64.0)	0.36
Female	8.0 (18.0)	4.0 (36.0)	
Median BMI, kg/m^2^ (IQR)	23.8 (22.1–26.8)	24.1 (22.6–26.9)	0.08
ASA Score			0.47
1	3.0 (6.0)	0 (0)	
2	39.0 (86.0)	10.0 (91.0)	
3	3.0 (6.0)	1.0 (9.0)	
Medical history			
Smoker	14.0 (31.1)	3.0 (27.0)	0.71
Hypertension	22.0 (48.8)	7.0 (64.0)	0.62
Cardiac disease	0	1.0 (9.0)	0.05
Diabetes	6.0 (13.3)	4.0 (36.0)	0.11
Surgical history	2.0 (4.4)	1.0 (9.0)	0.59
Laterality			0.31
Left	19.0 (42.0)	7.0 (64.0)	
Right	26.0 (58.0)	4.0 (36.0)	
Mass location			0.19
Upper	8.0 (18.0)	4.0 (36.0)	
Interpolar	35.0 (77.8)	5.0 (45.0)	
Lower	2.0 (4.4)	0 (0)	
Renal score			0.52
Mild complexity (4–6)	0 (0)	0 (0)	
Moderate complexity (7–9)	17.0 (37.8)	3.0 (27.3)	
High complexity (10–12)	28.0 (62.2)	8.0 (72.7)	

### Perioperative outcomes

**Figure 2:**
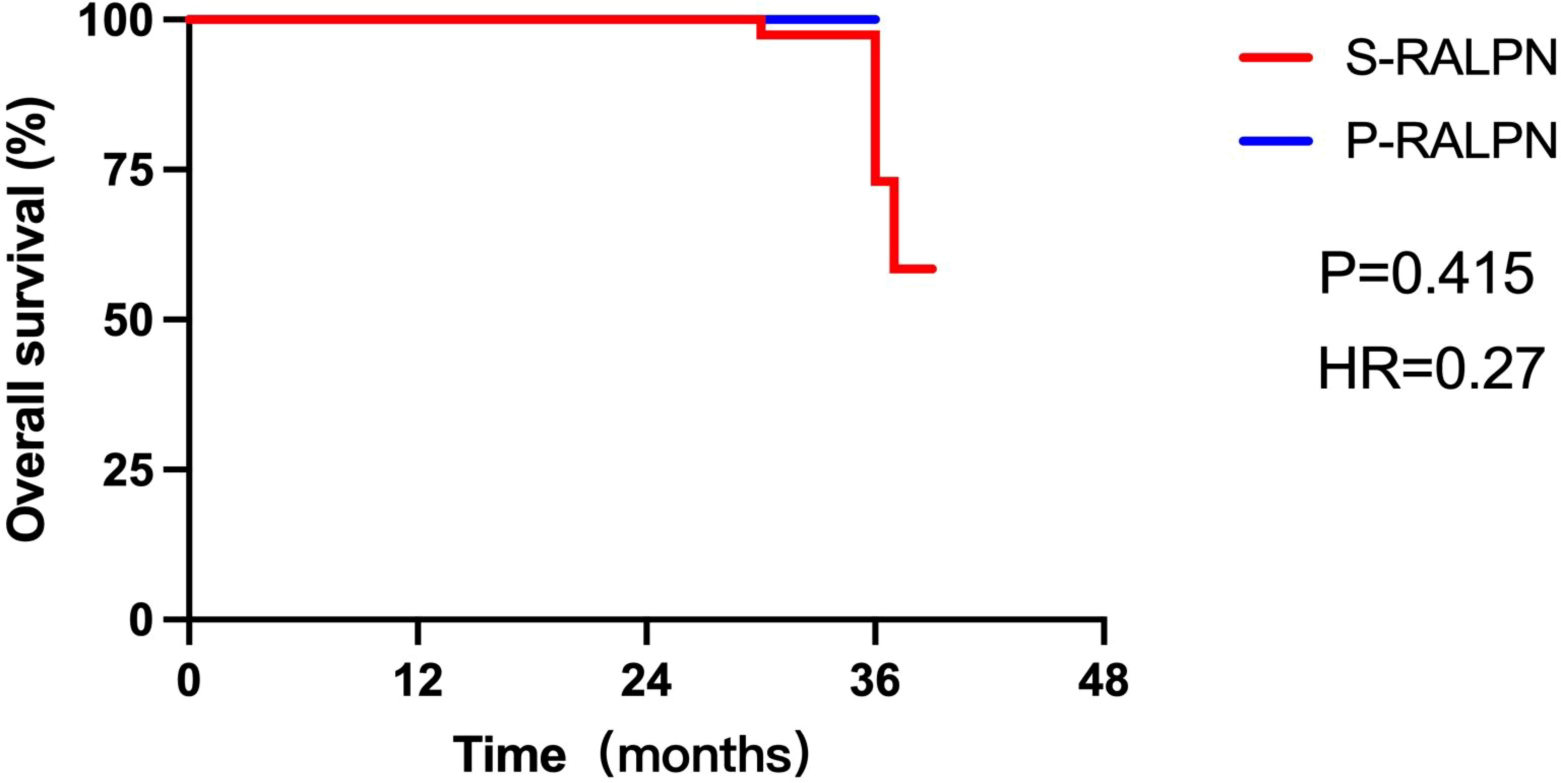
The schematic diagram of the S-RALPN.

The operative time of patients who received P-RALPN was slightly longer than those who received S-RALPN, but it did not reach statistical significance (125.9 versus 103.1 min; *p* = 0.09). Patients treated with P-RALPN had less intraoperative estimated blood loss than those treated with S-RALPN (81.8 versus 113.0 ml; *p* = 0.24). No patients received intraoperative blood transfusion in both groups. There were also no significant difference on the median postoperative hospitalization (5.9 versus 5.4 days; *p* = 0.59), exhaust time after surgery (2 versus 2 days; *p* = 0.94), and food-taking time (1.2 versus 1.1 days; *p* = 0.87) between P-RALPN and S-RALPN groups (**Table 2**). All patients underwent intraoperative fast frozen biopsy to check the pathology of the tumors. Most tumors showed malignancy during the surgery (89.2%). As for the Fuhrman grade, the pathological results of the tumors between the two groups were mainly distributed at levels 2 and 3, and no obvious difference was observed between P-RALPN and S-RALPN groups. Renal clear cell carcinoma is the main pathological types, with 33 cases in the S-RALPN group and 8 cases in the P-RALPN group. There was also no difference on the final pathological types between two groups (**Table 2**). After a median follow-up of 33.8 months in the S-RALPN group and 21.4 months in the P-RALPN group, patients had similar postoperative survival time between the two groups (hazard ratio = 0. 27, *p* = 0.42; **Figure 2**).

**Table 2:** Surgical and pathological outcomes.

	S-RALPN, *n* (%)	P-RALPN, *n* (%)	*p*
Median operating time, min (IQR)	103.1 (60.0–210.0)	125.9 (50.0–260.0)	0.09
Median estimated blood loss, ml (IQR)	113.0 (50–300.0)	81.8 (20.0–200.0)	0.24
Intraoperative transfusion	0 (0)	0 (0)	—
Median postoperative hospitalization, days (IQR)	5.4 (3.0–9.0)	5.9 (4.0–8.0)	0.59
Exhaust time after surgery	2.0 (1.0–4.0)	2.0 (1.0–3.0)	0.94
Food-taking time (day)	1.1 (1.0–3.0)	1.2 (1.0–2.0)	0.87
Conversion to open	0 (0)	0 (0)	—
Primary pathology (%)			
Benign	5.0 (11.0)	1.0 (9.0)	—
Malignant	40.0 (88.0)	10.0 (91.0)	—
Fuhrman grade			
1	4.0 (9.0)	0	—
2	22.0 (49.0)	5.0 (45.0)	
3	15.0 (33.0)	5.0 (45.0)	
4	4.0 (9.0)	1.0 (9.0)	
Secondary pathology (%)			
Clear cell	33.0	8.0	0.18
Angiomyolipoma	3.0	0	
Chromophobe	9.0	2.0	
Papillary	0	1.0	
Last follow-up, median (range), months	33.8 (24.0–39.0)	21.4 (11.0–36.0)	0.12

### Perioperative outcomes

In the P-RALPN group, the median volume of perfusion solution was 533.18 ml (255.0–750.0 ml). The median temperature of kidney surface was 22.64°C (21.7–24.1°C) after the kidney cools down. The median minimum intraoperative temperature of patients was 36.1°C (35.2–36.7°C). The pre-operative versus post-operative GFRs were comparable (median pre-operative versus post-operative GFR: 84.46 versus 66.10 ml/min). The median cold ischemia time was 33.0 min (25.0–39.0 min) (**Table 3**). In S-RALPN group, the median pre-operative versus post-operative GFR was 71.9 versus 43.0 ml/min. The median warm ischemia time was 21.5 min (21.0–30.0 min) (**Table 3**). The ischemia time in the S-RALPN group was significantly lower than it in P-RALPN group (21.5 versus 34.8 min; *p* < 0.01). However, the loss of GFR was much higher for the S-RALPN group after the surgery (28.9 versus 18.4; *p* < 0.01).

**Table 3:** Intraoperative data.

	S-RALPN	P-RALPN	*p*
Volume of perfusion solution/ml	—	533.2 (255.0–750.0)	—
Temperature of affected kidney/°C	—	22.6 (21.7–24.1)	—
Pre-operative GFR/(ml/min)	71.9 (34.94–133.86)	84.5 (34.81–123.05)	—
Post-operative GFR (ml/min)	43.0 (19.40–84.78)	66.1 (27.32–94.91)	—
GFR decline	28.9	18.4	<0.01
Minimum intraoperative temperature of patients/°C	—	36.1 (35.2–36.7)	—
Ischemia time/min	21.5 (21.0–30.0)	34.8 (30.0–39.0)	<0.01

## Discussion

Renal artery cold perfusion was the most common physiological form of renal hypothermia that has been used for many years (18, 19, 22). It could replace the residual blood of the kidney and rapidly cool the renal parenchyma. Moreover, it could prevent blood clotting in blood vessels, which allows for a clear surgical field of vision. Cold perfusion was used in kidney transplantation to preserve the organ and the body by OPN or LPN as well (23). The Ringer’s solution, instead of normal saline, was leveraged as perfused liquid because it could be used to adjust body fluids, electrolytes, and acid–base balance. Lactated Ringer’s solution is suitable for acidosis or dehydration cases prone to acidosis; thus, it was commonly used in operating rooms. A previous study suggested that it would cause irreversible renal ischemia–reperfusion injury (IRI) when the WIT of the affected side of the kidney was longer than 20 min (24). At present, there were few reports in previous studies on renal artery cold perfusion technology. In previous reports, renal hypothermia technology can effectively reduce renal parenchyma temperature to below 25°C and protect renal function when the WIT exceeds 30 min (18, 19). Hence, an experienced urological surgeon could suspect that the ischemia time would be more than 30 min and even much longer *via* clinical imaging diagnosis. The P-RALPN would be a safe choice.

The P-RALPN has many advantages: (i) It reduces the time of warm ischemia. The kidney is extremely sensitive to warm ischemia. Literature reported that the warm ischemia period should not exceed 30 min during partial nephrectomy (25–27). The energy-consuming metabolic activity of cortical cells reduces the consumption of oxygen and ATP, protects renal function, and prolongs the operation time. (ii) It saves time looking for the renal artery and blocking it. After cooling the kidney, the operator injected a 1.5-cm water balloon into the renal artery balloon *in vitro* to start blocking the renal artery. If the color of the kidney surface turns to white and ultrasound confirmed that the blood flow was stopped, then the renal artery was successfully blocked. This could save time in separating tissue to find and block the renal artery during operation. (iii) The blockage of the balloon catheter in the renal artery will not cause hemostatic clips to fall off. If the tumor was mainly supplied by one branch, the balloon can be placed in the feeding artery. When the renal artery has multiple branches, intraoperative clamps can be used to block the remaining branches. (iv) Although a large amount of hypothermic perfusion fluid entering the systemic circulation may lead to hypothermia and electrolyte imbalance, after rapid intraoperative perfusion, renal tumor resection is performed immediately. Power system control can also prevent a large amount of perfusate from entering the systemic circulation due to operational errors. (v) It offers a better field of vision (3D surgical field) to the surgeon with the assistance of the Da Vinci robot, which could provide a more precise and flexible operation in order to reduce the amount of intraoperative blood loss and protect renal function after operation. (vi) The application of a balloon catheter can provide renal hypothermia and renal artery occlusion for larger tumors or complete intrarenal tumors (21, 22, 28).

In the current study, P-RALPN showed limited damage on renal function. Moreover, the WIT during operation was more than 30 min. We conducted a detailed evaluation of the patients during operation. The catheter should be placed as far as possible in the distal end of the renal artery when performing renal artery balloon catheterization, which could avoid easy displacement or insufficient balloon occlusion. Furthermore, any voluntary body displacement should be leveraged to avoid blockage of the extracorporeal catheter. In our study, catheter placement was successfully performed in all 11 patients, and there was no blockage or catheter displacement. Moreover, we have summarized the preliminary experience in the process of cold perfusion of the kidney. First, perfusion with a fast flow rate (100 ml/min) into the affected kidney can minimize the impact on the patient’s body temperature and the body’s electrolyte balance. The temperature of the affected kidney is lowered, followed by perfusion at a slow flow rate (30 ml/min) to maintain the kidneys in a state of cold ischemia and low metabolism. The perfusion rate can be adjusted according to the real-time surface temperature of the affected kidney. Although P-RALPN had significantly higher ischemia time S-RALPN, the loss of GFR was much higher for the S-RALPN group after surgery (28.9 versus 18.4; *p* < 0.01). Hence, these results reveal that P-RALPN is more effective than S-RALPN. P-RALPN as a rare alternative option of tumor enucleation could be deeply considered in more complex and huge renal tumors. In the past, several studies revealed that parenchymal excision and damage were minimized to preserve renal function. However, it was difficult to complete tumor enucleation and renal parenchymal preservation in a short time, especially for a huge volume regardless of tumor location. P-RALPN could effectively prolong the ischemia time, thus preserving kidney function (29, 30).

Nevertheless, this novel surgical approach had several limitations that should be acknowledged. First, the operation time is relatively long and the process seemed to be more complicated. This is due to the pre-operative renal artery catheterization in the intervention room, intraoperative transport, and postoperative pressure hemostasis at the puncture point. Second, this is a retrospective study with a limited sample size. The current results should be interpreted with caution. There was no long-time renal function measurements in our study. A future investigation with a large sample size and a long follow-up is warranted. Third, it is hard to standardize the surgical options based on tumor complexity, leading to inevitable selection bias. Finally, P-RALPN costs slightly more than S-RALPN since the patient needs to undergo renal artery catheterization.

## Conclusion

For patients with complex renal tumors, P-RALPN showed similar intraoperative estimated blood loss, postoperative hospitalization, and postoperative survival time to S-RALPN, suggesting that P-RALPN is a safe and feasible surgery in the treatment of patients with complex renal tumors. Moreover, when the surgical proficiency eventually improves, the ischemia time of P-RALPN will decrease and the benefits will be greater.

## Data availability statement

The original contributions presented in the study are included in the article/supplementary material. Further inquiries can be directed to the corresponding authors.

## Ethics statement

All study participants were informed about the planned procedure and signed informed consent. The study was approved by the ethics committee of Zhejiang Provincial People’s Hospital, Hangzhou city, China (2022QT005).

## Author contributions

FL, QZ, and YB: Project development. YB, YKY, and FW: Manuscript writing. JQ, YY, QZ, and FW: Data collection and analysis. All authors read and approved the final manuscript.

## Funding

This study was supported by the Health Commission of Zhejiang Province (Grant No. 2021439557). This project was funded by Zhejiang Medical and Health Science and Technology Project (Grant No. 2019RC110).

## Conflict of interest

The authors declare that the research was conducted in the absence of any commercial or financial relationships that could be construed as a potential conflict of interest.

## Publisher’s note

All claims expressed in this article are solely those of the authors and do not necessarily represent those of their affiliated organizations, or those of the publisher, the editors and the reviewers. Any product that may be evaluated in this article, or claim that may be made by its manufacturer, is not guaranteed or endorsed by the publisher.

## Abbreviations

P-RALPN, Perfusion by renal artery balloon catheter during robot-assisted laparoscopic partial nephrectomy; S-RALPN, Standard robot-assisted laparoscopic partial nephrectomy; R.E.N.A.L. score, A Comprehensive Standardized System for Quantitating Renal Tumor Size, Location and Depth, consisting of radius (tumor size as maximal diameter), exophytic/endophytic properties of the tumor, nearness of the tumor’s deepest portion to the collecting system or sinus, anterior (a)/posterior (p) descriptor, and the location relative to the polar line; OPN, Open partial nephrectomy; LPN, Laparoscopic partial nephrectomy; IRI, Ischemia–reperfusion injury; WIT, Warm ischemia time; ASA, American Society of Anesthesia; BMI, Body mass index; GFR, Glomerular filtration rate.
